# The Origins, Specificity, and Potential Biological Relevance of Human Anti-IgG Hinge Autoantibodies

**DOI:** 10.1100/tsw.2011.107

**Published:** 2011-05-26

**Authors:** Randall J. Brezski, David M. Knight, Robert E. Jordan

**Affiliations:** Biologics Research, Centocor R&D Inc., Radnor, PA, USA

**Keywords:** autoantibodies, antibody-dependent cellular cytotoxicity, complement, monoclonal antibodies

## Abstract

Human anti-IgG hinge (HAH) autoantibodies constitute a class of immunoglobulins that recognize cryptic epitopes in the hinge region of antibodies exposed after proteolytic cleavage, but do not bind to the intact IgG counterpart. Detailed molecular characterizations of HAH autoantibodies suggest that they are, in some cases, distinct from natural autoantibodies that arise independent of antigenic challenge. Multiple studies have attempted to define the specificity of HAH autoantibodies, which were originally detected as binding to fragments possessing C-terminal amino acid residues exposed in either the upper or lower hinge regions of IgGs. Numerous investigators have provided information on the isotype profiles of the HAH autoantibodies, as well as correlations among protease cleavage patterns and HAH autoantibody reactivity. Several biological functions have been attributed to HAH autoantibodies, ranging from house-cleaning functions to an immunosuppressive role to restoring function to cleaved IgGs. In this review, we discuss both the historic and current literature regarding HAH autoantibodies in terms of their origins, specificity, and proposed biological relevance.

## INTRODUCTION

A long-standing question in immunology is how individuals are capable of generating a very broad range of diversity against foreign antigens, while at the same time eliminating reactivity against self-antigens. Autoreactive B cells are regulated by tolerance checkpoints during B-cell development, which can occur in the bone marrow (central tolerance) or in the periphery (peripheral tolerance). B cells recognize antigen through their B-cell antigen receptors (BCR), which can subsequently be secreted as antibodies into the serum as a part of the humoral immune response. When a breakdown in either B-cell central or peripheral tolerance occurs, individuals can generate autoantibodies directed against self-antigens. In some cases, autoantibodies are thought to correlate with pathological conditions attributed to autoimmune dysfunction, such as autoimmune hemolytic anemia, myasthenia gravis, and idiopathic thrombocytopenic purpora (ITP). However, most healthy individuals have circulating B cells that recognize numerous self-antigens. Several types of autoantibodies have been characterized, including those directed against immunoglobulins. Humans have five classes of immunoglobulins: IgM, IgD, IgG, IgA, and IgE. The best characterized anti-immunoglobulin autoantibodies are those directed against the Fc region of IgG, known as rheumatoid factor, or those directed against the variable regions, known as anti-idiotype autoantibodies. Over the years, another class of anti-immunoglobulin autoantibody has been described: those that bind specifically to proteolytically exposed epitopes containing C-terminal amino acid residues in either the upper or lower hinge region. These human antihinge (HAH) autoantibodies generally are not detected as binding to the intact IgG counterpart.

The origin of HAH autoantibodies is not entirely clear. They are sometimes referred to as natural autoantibodies (NAbs). There are at least two broad definitions attributed to the term NAbs. One definition indicates that they arise independent of an immune response[1] and contain variable regions that are comprised of germline-encoded genes[2]. The other definition refers to the subset of low-affinity (and sometimes polyreactive) autoantibodies that are present at a steady-state level in otherwise healthy individuals[3]. Variable region cloning and analysis could provide some clues as to the origin of antihinge autoantibodies. To our knowledge, only two antihinge autoantibodies have been cloned. The heavy-chain variable regions of those two HAH autoantibodies had 88% homology with the nearest germline sequence[4], suggesting that somatic hypermutation had occurred during an immune response[5]. More recent studies have shown that HAH autoantibodies exist that react with a wide range of epitopes[6], suggesting that the two cloned antihinge autoantibodies may not be fully representative of the entire repertoire of HAH autoantibodies.

The biological function of autoantibodies directed against cleaved IgGs is enigmatic. The presence of antihinge-specific autoantibodies was first detected in rabbits in the 1960s when serum factors were described that bound to determinants exposed following the cleavage of polyclonal rabbit autoantibodies with either papain or pepsin[7,8]. Subsequent studies in the human system not only uncovered similar specificities, but were extended to investigations of their presence in a number of disease states, including cold agglutination (CA)[9], human immunodeficiency virus (HIV)[10], rheumatoid arthritis[11], and systemic lupus erythematosus (SLE)[12]. Several investigations suggested that HAH autoantibodies could play an immunosuppressive role by binding to the hinge region of antigen-engaged B cells and inducing apoptosis via coaggregation of the BCR with FcγRIIb (reviewed in Terness et al.[5]). Other proposed functions include complement amplification[13] or providing a surrogate Fc domain to cleaved IgGs to restore Fc-mediated effector functions, such as antibody-dependent cellular cytotoxicity (ADCC) and complement-dependent cytotoxicity (CDC)[6].

In this review, we will discuss each of the above-mentioned aspects of HAH autoantibodies, spanning the early literature to the more recent findings. Because a substantial fraction of the literature reviewed is historic, and cellular and molecular biological techniques have advanced considerably, we will also point out where new approaches could potentially shed light on the origins, specificity, and potential biological functions of HAH autoantibodies.

## B-CELL DEVELOPMENT AND AUTOANTIBODY GENERATION

In mice, there are at least three unique B-cell lineages: B1a, B1b, and B2[14]. B1a B cells develop from precursors in the fetal liver and are thought to contribute to natural IgM antibodies found in the serum. The variable regions of B1a B cells are nonmutated. B1b B cells originate from precursors in the bone marrow. In contrast to B1a B cells, B1b B cells can express mutated IgG variable regions[15]. Both B1a and B1b B cells reside in the pleural and peritoneal cavities, are capable of responding to T-cell–independent antigens, and participate in the first line of defense against foreign pathogens[16]. B2 B cells develop from precursors in the bone marrow throughout life; they are capable of responding to T-cell–dependent antigens and are considered an essential part of the adaptive immune response. At present, B1 B cells are less well characterized in humans[1,17].

In both humans and mice, B-cell development is characterized by an ordered and sequential assembly of the BCR, beginning with assembly of the preBCR followed by the mature BCR. The mature BCR is a multisubunit, transmembrane protein consisting of an immunoglobulin heavy chain (IgHC) covalently linked to an immunoglobulin light chain (IgLC) by disulfide bonds. The IgHC/IgLC heterodimer is noncovalently linked to the signaling subunits Igα and Igβ. Expression of the IgHC is primarily regulated by a process known as V(D)J recombination, which also contributes to the initial generation of BCR diversity. During this process, gene segments known as the variable (V), diversity (D), and joining (J) regions recombine to form a series of unique, heavy-chain variable regions. Expression of the IgLC is regulated by a similar process involving recombination of the light-chain V and J gene segments. These processes result in the broad diversity of antigen recognition that allows B cells to cope with the myriad of foreign invaders encountered over the lifetime of the host. One potentially problematic consequence of IgHC and IgLC recombination is that the gene rearrangements occur indiscriminately, meaning that antigen recognition sites can recognize both foreign antigens and self-antigens. It was estimated that early immature B cells have approximately 75% self-reactive BCRs, and those BCRs were polyreactive against antigens such as insulin, DNA, and lipopolysaccharides (LPS)[18]. Additionally, many of the self-reactive BCRs recognized antigens from the nucleus[18]. Most of these self-reactive B cells were eliminated in the bone marrow by the first tolerance checkpoint. Three main processes have been identified that are responsible for eliminating self-reactive B cells. First, bone marrow and early-stage peripheral autoreactive B cells that recognize self-antigen can be deleted by apoptosis, a process known as negative selection[19,20]. Second, autoreactive B cells that receive intermediate signals can undergo additional recombination events, known as receptor editing[21]. Third, self-reactive B cells can enter a state of unresponsiveness, termed anergy[22]. It was estimated that the majority of self-reactive cells are eliminated by receptor editing[23].

One way in which B cells can escape peripheral tolerance is during the natural progression of an immune response[24]. Mature, naive B cells that initially become activated in an immune response are thought to undergo several different fate paths[25,26,27]. A subset of antigen-challenged B cells rapidly differentiates into short-lived immunoglobulin-secreting plasma cells (PC), which contribute to the initial defense against foreign pathogens. Another subset of activated B cells migrates to B-cell follicles to produce germinal centers, which aids in the development of serological memory by production of memory B cells and long-lived PC. One process that contributes to humoral memory is class-switch recombination, whereby the immunoglobulin constant regions switch to other immunoglobulin isotypes, such as IgG. Another process is somatic hypermutation, which entails introduction of random gene modifications within the IgHC and IgLC variable regions. As a result of these two processes, B cells with new variable regions and isotypes are selected within the germinal center reaction. A recent study has indicated that a significant increase in self-reactive IgG+ memory B cells emerge out of an immune response, suggesting that *de novo* autoreactive B cells can result from the process of somatic hypermutation[28].

## ORIGINS OF HAH AUTOANTIBODIES

The mechanism that results in the production of HAH autoantibodies is not entirely clear and any association with chronological age has not been studied to our knowledge (i.e., their presence or absence in young children). However, the presence of HAH autoantibodies can be detected to varying degrees in most healthy adults[6]. Therefore, it seems likely that individuals expressing HAH autoantibodies could have populations of circulating B cells in the periphery that have HAH-reactive BCRs. In investigations on the antigen binding domain of HAH autoantibodies, Welschof and colleagues provided data to confirm this supposition. The authors constructed a phagemid library from the peripheral blood mononuclear cells (PBMCs) isolated from an individual that had high anti-$F(ab')_2$ titers and were able to clone out two anti-$F(ab')_2$ autoantibodies[4]. The study did not evaluate the cell surface marker phenotype of the anti-$F(ab')_2$–expressing B cells from which the phagemid library was generated. However, the cloned variable regions provided clues about the origin of those two anti-$F(ab')_2$ autoantibodies. The two IgHC variable regions had 88% homology with the closest germline sequences. In contrast, the two IgLC variable regions were nearly identical to the closest germline sequences (100 and 99.3% homology). Due to the fact that the two cloned IgHC variable regions from anti-F(ab')$_2$–expressing B cells had 88% homology with the closest germline regions, it seems unlikely that these clones derived from NAb-expressing B cells. As previously noted, two hallmarks of memory B cells are class-switch recombination and somatic hypermutation, characteristics displayed by both of the cloned anti-F(ab')$_2$ autoantibodies. Therefore, it is possible that these two clones were derived from memory B cells.

Perhaps additional clues into the origins of HAH B cells can be inferred from the isotype of HAH autoantibodies. A recent study conducted in our lab indicated that HAH autoantibodies display a diverse number of immunoglobulin isotypes[6]. We found HAH autoantibodies of the IgM, IgG1, IgG2, IgG3, and IgA isotypes, with a particular emphasis on the IgG3 isotype. The prevalence of HAH autoantibodies of the IgG3 isotype has been noted by other labs as well[11]. Although class-switch recombination and somatic hypermutation have been documented to occur outside of germinal centers[29,30], it is possible that HAH autoantibodies with isotypes other than IgM could have occurred as a result of a germinal center reaction. It is also important to note that a significant component of HAH autoantibodies was found to be of the IgM isotype, which could perhaps represent antihinge NAbs. Taken together, since HAH autoantibodies have been identified that react with a wide range of IgG hinge epitopes with C-terminal exposed amino acid residues, and contain a number of different isotypes, HAH autoantibodies could potentially develop from several distinct B-cell lineages. Therefore, the overall repertoire of HAH autoantibodies could include NAbs as well as independent HAH autoantibody clones that developed from an adaptive immune response.

The advent of multiparameter flow cytometric analysis has afforded investigators the ability to not only isolate antigen-specific B cells, but also to phenotype the lineage of those B cells by analyzing cell surface markers. Additionally, the use of single-cell sorting technologies has proven to be a useful tool for the cloning of antigen-specific B-cell variable regions[31]. Future studies in which the variable regions of HAH autoantibodies can be cloned, combined with cell surface marker phenotype analyses, will lead to further insights into the genetic diversity of this population of autoantibodies.

## SPECIFICITY OF HAH AUTOANTIBODIES

Following the demonstration that rabbit IgGs could be proteolytically fragmented by papain to yield Fab fragments[32] and by pepsin to yield F(ab')$_2$[33], it was observed that new antigenic determinants were exposed that were recognized by pre-existing autoantibodies in rabbit serum[7,8]. Moreover, different populations of rabbit autoantibodies that recognized Fab and F(ab')$_2$, but that showed no similar reactivity to intact rabbit IgG, were found. These insightful early observations (obtained with polyclonal rabbit antigen fragments and autoantibodies, and with the agglutination methods then employed) prompted similar human investigations.

The utility of papain and pepsin was applied to the generation of Fab and F(ab')$_2$ fragments from human IgGs. Two Fab fragments are released from IgG when the heavy chains are each cleaved in the upper hinge, an event that simultaneously generates an Fc fragment in which the two heavy-chain components remain covalently attached through the disulfide linkages of the core hinge (e.g., with papain). F(ab')$_2$ fragments result from two separate peptide bond cleavages in the lower hinge, leaving the two Fab domains linked through the core disulfides (e.g., with pepsin). As in the rabbits, human serum autoreactivity to the IgG fragments was readily detected and designated as papain agglutinators[34,35,36] or pepsin agglutinators[37] by some authors to denote their capacities for immunoprecipitation of the respective fragments. This topic was actively investigated, and numerous additional groups contributed to the characterization and specificity of human antifragment autoantibodies[35,38,39,40]. Investigations were also performed on IgGs cleaved with different enzyme sources. Bacterial proteases (subtilisin, bromelain), human digestive enzymes (trypsin, pancreatic elastase, and chymotrypsin, in addition to pepsin), plant-derived proteases (ficin and papain), and the human fibrinolytic protease, plasmin, were all found to yield IgG fragments that were recognized by apparently distinct populations of human serum antibodies[41,42,43]. A consensus emerged that antifragment reactivity was widespread in the human population[44,45], although of generally low affinity[46] and with considerable heterogeneity in titer among different individuals[46,47]. It was commonly observed that an individual's antifragment autoantibodies showed more reactivity to $F(ab')_2$ than to Fab fragments[38,48].

The majority of the early investigations focused on papain- and pepsin-digested polyclonal IgGs, and these sometimes led to conflicting interpretations regarding whether autoantibody antifragment reactivity was directed to hinge epitopes or was directed to idiotypic or framework determinants[35,49]. Even as methodologies transitioned to more sensitive radioimmunoassays (RIA)[45,49] and enzyme-linked immunosorbent assay (ELISA) formats[36,47], ambiguities regarding binding specificity remained due to the polyclonal nature of the reagents employed. Indeed, one study employing epitope-mapping approaches concluded that certain epitopes for autoantibody recognition resided within the variable and constant regions of λIgLCs[50]. We are not aware of other studies that point to IgLC epitopes for autoantibody recognition. Concerns about the physiological relevance of autoantibodies directed to new antigenic epitopes generated with papain and pepsin derived from the nature of these enzymes themselves. Papain (vegetable derived) and pepsin (active in acidic stomach conditions) do not normally reach human nondigestive tissues or circulation. However, the peptide bonds cleaved by papain and pepsin might reflect parallel specificities with other, more physiologically relevant, proteases to thereby reveal analogous epitopes for autoantibody recognition. However, early detailed correlations of autoantibody binding and cleavage site specificity were seldom possible, due at least in part to the polyclonal and multi-isotype nature of human IgG preparations. Nevertheless, the improved assay formats did afford better descriptions of human antifragment autoantibodies, and prompted a number of queries regarding associations to human immune responses and pathologies (described earlier in this review). As also mentioned previously, some studies pointed to unexpected serological findings, including that antihinge autoantibodies were biased toward minor isotypes, including IgG3[11].

Recent investigations of antihinge autoantibodies have been facilitated by the use of monoclonal antibodies and peptide analogs of specific hinge sequences, as well as the deployment of physiologically relevant proteases for generation of the IgG fragments. The earlier studies in almost all cases treated Fab and $F(ab')_2$ as generic entities without a recognition that subtly different fragment lengths result from different protease cleavage sites. We and others found that a number of human and bacterial proteases cleaved human IgG1 within the upper and lower hinge domains[51,52,53,54] and defined a number of different cleavage points therein (Fig. 1). Almost without exception, protease cleavage of IgGs has been assessed in solution in purified systems, a set of conditions that may not always be analogous to those *in vivo*. In any case, it became evident that the different proteolytically generated Fab and $F(ab')_2$ fragments elicited varying degrees of autoantibody recognition, and in some cases, the reactivity was higher than that seen with the fragments generated with papain and pepsin[6].

With many of the human physiologic proteases, IgG cleavage was observed to proceed more slowly compared to papain and pepsin. However, the natural roles of papain (defense against fungal or microbial attack on the papaya plant) and pepsin (rapid digestion of protein in the stomach) are likely unrepresentative of the actions of extracellular proteases in human tissue. Moreover, the human extracellular proteases that have been described to cleave IgGs are better recognized for their natural roles in physiology or pathology (e.g., the metalloproteinases from immune cells and tumor cells work to break down extracellular matrix components during migration and invasion[55,56]). Nevertheless, a number of such proteases have now been shown to cleave IgG and, in many cases, with well-defined specificity within the hinge. The generally slower rates of solution-phase IgG cleavage compared to papain and pepsin may have hindered appreciation for the potential impact of physiological IgG-degrading enzymes in pathological settings. One bacterial protease, IdeS from *Streptococcus pyogenes*, is rapid acting and is remarkably specific for cleavage of the IgG lower hinge[54]. Taken together, the cumulative evidence led us to further speculate that host immune defenses against invasive cells (tumors, bacteria) may be compromised by local protease expression[57].

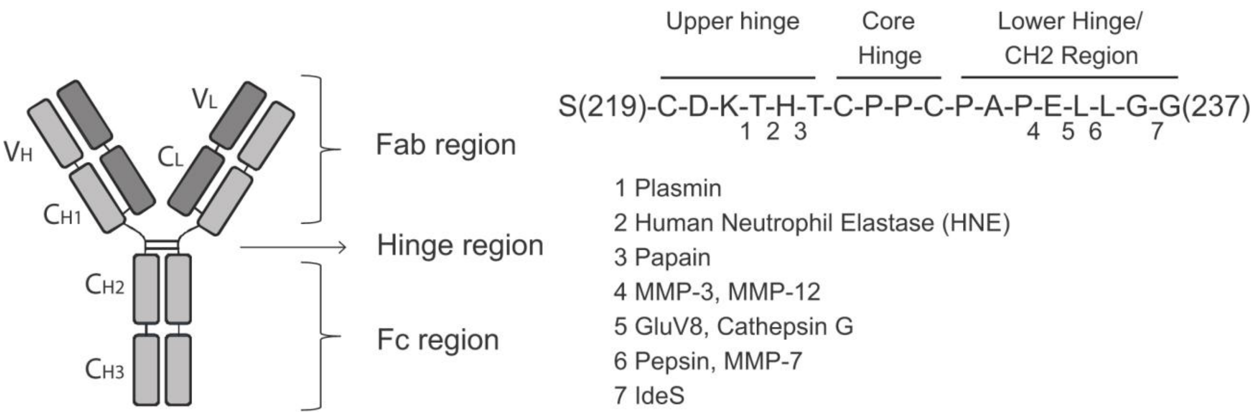

**FIGURE 1.** A schematic representation of a human IgG1 antibody (left side) and the amino acid sequence of the hinge region (right side). The numbers below the amino acid sequence indicate specific protease cleavage points within the hinge region.

To test whether endogenous proteolysis of IgG could be correlated with autoantibody reactivity to revealed epitopes containing C-termini, it was desirable to obtain independent assessments of binding to different potential positions of cleavage within the hinge. Peptide analog approaches have been variously adopted to define the fine specificity of antihinge antibodies[6,48,58,59]. In one study[48], human serum antihinge antibodies were prepared by affinity isolation on F(ab')$_2$ fragments generated with pepsin (the cleavage site in IgG1 assigned between L234 and L235)[48,60,61,62]. The autoantibodies were tested for binding to two different two-chain peptide analogs, terminating in the IgG1 lower hinge “TCPPCPAP” and the longer “TCPPCPAPELLGG” (note that the autoantibodies were isolated on the basis of their affinity to an IgG fragment with a C-terminal cleavage site intermediate to the termini of these two peptides). The test peptides were dimeric (in accord with the actual hinge in intact IgG1), possessed the core disulfide-linked hinge structure, and displayed unmodified N- and C-termini. ELISA binding of human serum samples showed strong binding to the longer dimeric peptide and less to the shorter (and almost no binding to the single-chain peptide). The authors concluded that the polyclonal autoantibody preparations bound to a conformational epitope requiring the (disulfide-linked) core hinge.

Subsequent studies from the same group advanced their interpretation of the core hinge being a component of a discontinuous, conformational epitope for antihinge autoantibody binding. As previously noted, Welschof and colleagues cloned two HAH antibodies with affinity for human F(ab')$_2$ fragments (pepsin generated)[4]. The two antibodies were expressed as single-chain Fv constructs and were extensively characterized with regard to binding to dimeric hinge peptide analogs in which each position (except cysteines) was substituted by every other amino acid[58]. The cloned autoantibodies bound to the wild-type hinge sequence and exhibited tolerance for many substitutions in most hinge positions, with the exception of three proline residues that appeared essential to the binding by these scFvs. It should be noted that the double-stranded peptides could be considered analogs of proteolytically generated Fc fragments with a free N-terminus at T225 in the upper hinge, a departure from earlier peptide studies incorporating a free C-terminus or the more historical approaches using Fab or F(ab')$_2$ fragment analogs with free C-termini. Nevertheless, the results were consistent with the core hinge, constituting a component of the epitope for these particular autoantibodies.

Studies from our own laboratory focused on HAH autoantibodies that bound to hinge structures conforming to epitopes expected to be present on Fab and F(ab')$_2$ fragments. We adopted an epitope-mapping approach in which the selectivity of autoantibody binding was presumed to reflect differences in the carboxy terminus of the proteolytically generated fragments[6]. To address this, progressive single-chain, 14-mer peptides with an N-terminal biotin adduct and with free C-termini at each position between D221 and L251 were individually captured on streptavidin-coated microtiter wells in ELISA. We confirmed that reactive autoantibodies were often IgG3s, a finding that focused attention on this isotype for the subsequent investigations[6]. IgG3 autoantibodies from human serum showed a discontinuous pattern of binding reactivity for peptides with different C-termini in the IgG1 hinge[6]. First, no autoantibody reactivity was found for peptides terminating within the core "CPPC" sequence, suggesting that this component was either not sufficient to define an epitope or was comprised with amino acid positions that do not normally get exposed as antigens. Moderate levels of IgG binding were directed to peptide analogs in the upper hinge with termini at K222, T223, and H224 (analogs of sites of cleavage by plasmin, neutrophil elastase [greatest reactivity], and papain, respectively). Contrastingly, robust autoantibody binding to lower hinge analogs with C-terminal positions between P232 and F241 was found. This sequence encompasses points of cleavage by several human metalloproteinases, human cathepsin G, (porcine) pepsin, the *Staphylococcus aureus* GluV8 protease, and the *Streptococcus pyogenes* IdeS protease[53]. Antigenic peptides with intervening termini that are not identified as sites of protease cleavage also exist within this sequence and could represent sites of overlapping specificities to adjacent, known protease sites or may correspond to cleavage sites by yet unidentified enzymes.

It was noted that single-chain 14-mer peptide analogs of the same hinge region, but containing each position as a free N-terminus (and with the C-terminus blocked), yielded negligible autoantibody binding[63]. Thus, peptides with a particular amino acid as a C-terminus were often recognized by autoantibodies, whereas peptides originating from the opposite direction with the same amino acid as a free N-terminus were not bound. The reason for this disparity in antigenicity at the same site, but with opposed directionality, is unclear. Other investigators found a class of autoantibodies with specificity for a conformational hinge epitope that incorporated the disulfide-linked core and without regard to the orientation of the cleavage point[48]. We did not incorporate the core hinge in our epitope-mapping strategy, but instead showed specificity for the C-terminus of the single-chain analogs of cleavage sites. These differences may be revealing in pointing out the potential for multiple autoimmune specificities.

Proteolysis of IgGs with a number of physiological proteases yielded intermediate products not usually observed with more rapidly acting papain and pepsin, and therefore not usually encountered in laboratory digestion reactions. Specifically, human and bacterial extracellular proteases have been shown to cleave IgG to $F(ab')_2$ in two steps[52,53,64,65,66]. The first step, to a single-cleaved intermediate in the lower hinge (without removal of the Fc region), was found to be more rapid *in vitro* than the second step[66], and single-cleaved intermediates were observed to accumulate in pathological settings *in vivo*[53,66]. The limited proteolysis and accumulation of intermediate digestion products *in vivo* may be relevant with regard to recent observations that single-cleavage versions of cell-targeting IgGs lose substantial, if not all, Fc-mediated effector functions related to target cell killing[66], and that the Fc-mediated functions of such species may be restored by the binding of HAH antibody preparations to cell-bound cleaved IgGs[6]. Single-clipped hinge domains of IgG (and perhaps especially those that remain bound to cell surfaces) might expose physiologically relevant epitopes to allow host immune recognition of otherwise cryptic hinge elements. It remains to be shown if physiological IgG cleavage can be correlated with autoantibody reactivity to exposed antigens. Also remaining to be investigated are any clinical effects or consequences to patients with autoimmune diseases following the administration of high doses of intravenous immunoglobulin preparations containing low, but detectable (<0.1%), amounts of these autoantibodies[6].

## BIOLOGY OF HAH AUTOANTIBODIES

Autoantibodies have been studied for decades and have been linked to normal, immunological, regulatory mechanisms, as well as to a variety of diseases, including autoimmune disorders, such as SLE, multiple sclerosis (MS), nephritis, and others. Autoantibodies whose targets are immunoglobulins comprise one class of autoantibodies that has been studied extensively. For convenience, these anti-immunoglobulin autoantibodies are often classified according to the domain of the antibody molecule recognized by the autoantibodies. Anti-immunoglobulins may target the Fc constant region (e.g., rheumatoid factors), or the

Fab or F(ab')$_2$ domains. Autoantibodies that recognize the Fab or F(ab')$_2$ domains can be further divided into anti-idiotypic antibodies that target the variable regions, and antibodies that target the constant regions (heavy-chain CH1, hinge, IgLCκ or λ) of the Fab or F(ab')$_2$ domains. The proteolytic methodologies cited above were developed to cleave antibody molecules and separate the domains, thus facilitating the characterization of anti-immunoglobulins. As detailed, HAH autoantibodies react with Fab or F(ab')$_2$ fragments of immunoglobulins (primarily IgGs), but not with the intact IgG molecules. Because, as discussed above, Fab or F(ab')$_2$ fragments contain many potential autoantibody targets other than amino acids exposed by proteolytic cleavage, attribution of biological properties to HAH autoantibodies must be contingent upon experimental demonstration that the hinge region is, in fact, the autoantibody target.

## STUDIES WITH ANTI-FAB/ANTI-F(AB')$_2$ AUTOANTIBODIES

Anti-Fab and anti-F(ab')$_2$ autoantibodies exist in most normal humans[67]. Early speculation on the possible role of these normal autoantibodies centered around clearance mechanisms to remove proteolyzed antibodies in a normal process of antibody catabolism[46], although such a house-cleaning role has not been directly demonstrated. Anti-Fab or anti-F(ab')$_2$ autoantibodies have been associated with a variety of diseases. Persselin and Stevens[11] studied anti-Fab antibodies in the sera of rheumatoid arthritis patients and found that the autoantibodies were generally elevated compared to normal sera and were predominantly minor isotypes (IgG3 and IgG4). The prevalence of minor isotypes is consistent with a number of other studies[6,10], but its biological significance remains to be delineated. Anti-F(ab')$_2$ autoantibodies were studied in CA disease, which is characterized by the presence of autoantibodies frequently directed against red blood cell (RBC) antigens[9]. An inverse correlation was noted between RBC autoantibodies and anti-F(ab')$_2$ autoantibodies; the authors argue for an important role of the anti-F(ab')$_2$ autoantibodies in the regulation of RBC autoantibody production in CA patients. In another disease associated with elevated autoantibodies, SLE, a similar inverse correlation was noted; in this case, between anti-DNA autoantibodies and anti-F(ab')$_2$ autoantibodies[12]. Patients with active SLE often have low serum anti-F(ab')$_2$ and patients with SLE in remission often have high levels of serum anti-F(ab')$_2$ autoantibodies. The authors suggested that serum anti-F(ab')$_2$ may be depleted as the result of increased anti-F(ab')$_2$–containing immune complex deposition in renal tissues. Consistent with this idea, enrichment of anti-F(ab')$_2$ autoantibodies was observed in renal biopsy tissues from patients with active SLE. Anti-Fab autoantibodies have also been studied in patients with acquired immunodeficiency syndrome (AIDS) and aids-related complex (ARC)[68]. Patients with AIDS/ARC had significantly higher anti-Fab autoantibodies than HIV-positive patients without AIDS/ARC or uninfected control subjects. A highly significant inverse association was found between anti-Fab autoantibodies and CD4+ T-cell counts. An association has also been observed between IgA anti-Fab autoantibodies and disease stage in head-and-neck cancer patients[69]. Patients with squamous-cell carcinoma (SCCHN) and adenoid cystic carcinoma (ACCHN) of the head and neck had higher levels of IgA anti-Fab autoantibodies than healthy controls. In patients with SCCHN, there was a direct correlation between disease stage and IgA anti-Fab activity. A strong association was also seen between anti-Fab autoantibodies and severe infections by various microorganisms[67]. Anti-Fab autoantibodies were highest in patients with septicemia due to Gram-positive cocci.

The studies noted above describe correlations between various diseases and anti-Fab or anti-F(ab')$_2$ autoantibodies, with little data addressing mechanisms to explain the role of autoantibodies in the initiation or maintenance of disease. Because precise epitope-mapping information on the targets of anti-Fab and anti-F(ab')$_2$ autoantibodies and specific monoclonal reagents were not yet available, these early correlative studies could not distinguish among the different categories of anti-Fab or anti-F(ab')$_2$ autoantibodies, so that any involvement of specific HAH autoantibodies in these reported studies was unclear.

In addition to disease associations, anti-F(ab')$_2$ autoantibodies have been implicated in immune regulatory mechanisms. In a series of reports, Terness and colleagues demonstrated a potential immunoregulatory role of human anti-F(ab')$_2$ autoantibodies[5]. They focused on nonidiotypic autoantibodies that could potentially react with various parts of the F(ab')$_2$ molecule, including the IgLC constant region, the CH1 IgHC region, and the hinge region. An Fc-dependent immunosuppressive function of the autoantibodies was demonstrated, and it was proposed that the autoantibodies may contribute to the termination of a successful antibody response and/or the control of autoreactive B-cell clones**.** One suggested mechanism for the immunosuppressive effect involves binding of IgG anti-F(ab')$_2$ to the immunoglobulin-containing membrane BCR via the autoantibody Fab region, followed by cross-linking of the BCR and the inhibitory FcγRIIb receptor on the surface of B cells via the Fc region of the anti-F(ab')$_2$ autoantibody. Cross-linking the BCR and FcγRIIb can lead to apoptosis of the B cell, resulting in an immunosuppressive effect (Fig. 2A). In the simplest version of this model, the anti-F(ab')$_2$ targets on the membrane immunoglobulin molecule are constant-region determinants accessible on the immunoglobulin surface (e.g., IgHC CH1 or IgLCλ or κ). For HAH autoantibodies (whose targets are exposed upon proteolytic cleavage of IgG) to mediate suppression in this model, there must be a mechanism to reveal HAH epitopes on the membrane immunoglobulin since HAH autoantibodies do not bind to intact immunoglobulin. To accommodate the possibility of HAH involvement, the authors postulate that upon antigen binding to the Fab region of the membrane immunoglobulin portion of the BCR, a conformational change is transmitted to the hinge region, exposing specific HAH epitopes and allowing HAH binding with subsequent cross-linking to FcγRIIb. Consistent with this idea, selective inactivation of B cells has been shown when the BCR is occupied with antigen[70]. These studies showed that anti-immunoglobulin antibodies can suppress B-cell antibody production in an Fc-dependent manner, although it was not shown directly that HAH autoantibodies alone can mediate the suppression. A direct role for HAH autoantibodies remains to be demonstrated using hinge-specific reagents[48,53,58].

## STUDIES WITH DEFINED HAH AUTOANTIBODIES

Precise identification of hinge-region epitopes for HAH and development of highly specific detection reagents (see specificity section) has led to the identification of HAH autoantibodies as predictors of graft outcome in kidney transplantation[71,72,73]. Kidney graft recipients with a high pretransplant HAH titer of the IgG class had a significantly better survival rate than patients with low IgG HAH autoantibodies; a similar correlation was found in patients with high pretransplant HAH titers of the IgA class. Those patients with both high IgG and IgA HAH autoantibodies had the best survival rate. The basis of these correlations is not known, but may be due to an immunosuppressive effect (see above) of the HAH autoantibodies[71].

A role for HAH autoantibodies (which the authors refer to as antihinge NAbs) in complement amplification has been elucidated with a combination of *in vitro* and *in vivo* studies[13]. Well-characterized antihinge NAbs were shown to participate in the formation of immune complexes with antigen-bound F(ab')$_2$ fragments that can stimulate complement amplification via capture of dimeric C3b, known as a potent C3 convertase precursor (Fig. 2B). The authors suggested that binding of antihinge NAbs to the F(ab')$_2$ antigen complexes stabilizes the hinge region of the F(ab')$_2$ molecules, and facilitates binding of dimeric C3b to the known C3b binding site[74] in the CH1 region of the F(ab')$_2$ fragments. Alternatively, it may be that the antihinge NAbs themselves bind dimeric C3b, leading to active complement amplification complexes. Excessive complement amplification may trigger the systemic inflammatory response syndrome (SIRS) following microbial infection. Fumia and colleagues hypothesize that SIRS may be triggered by F(ab')$_2$ fragments generated from IgG molecules via proteolytic cleavage, by enzymes released from activated neutrophils or by proteases secreted by pathogens[13]. The F(ab')$_2$ fragments may then form immune complexes with antigens from the infecting pathogen and antihinge NAbs that recognize the F(ab')$_2$ hinge region. These complexes can then bind dimeric C3b, generating complexes that can stimulate complement amplification leading to SIRS.

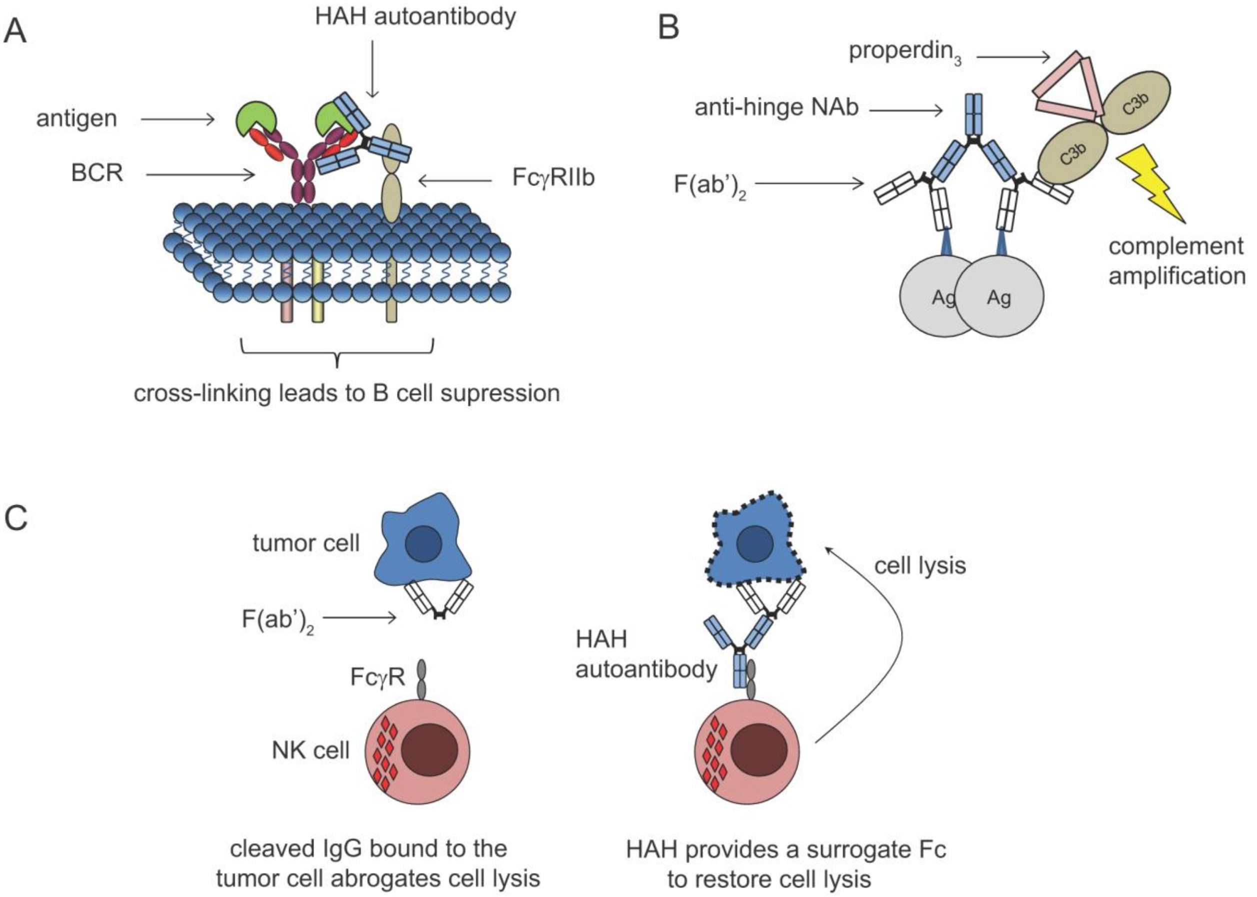

**FIGURE 2.** Proposed functional mechanisms for HAH autoantibodies. (A) The Fc region of autoantibodies that bind to antigen-engaged BCR coligate FcγRIIb, which leads to B-cell suppression (reviewed in Terness et al.[5]). (B) Antihinge NAbs that bind to immune-complexed $F(ab')_2$ fragments stiffen the hinge region of the $F(ab')_2$, resulting in docking of C3b and complement amplification[13]. (C) HAH autoantibodies bind to $F(ab')_2$ fragments on target cells and provide a surrogate Fc domain that can interact with effector immune cells, resulting in target cell destruction[6].

## POSSIBLE ROLE OF HAH AUTOANTIBODIES IN HOST DEFENSE

HAH autoantibodies have been identified in the sera of most normal humans[6,75], raising questions about their origin and functional significance:

1. How and where are the HAH targets (i.e., proteolyzed antibodies) generated *in vivo*?
2. What proteases are responsible?
3. What is the normal role(s) of HAH?
4. Are they involved in protection against diseases?

The function(s) of HAH autoantibodies remains to be established. In addition to a possible regulatory role in immunosuppression (described above), it has been suggested that HAH autoantibodies may play a role in augmenting host defenses against inflammatory and/or invasive diseases[57]. The targets of HAH autoantibodies (i.e., fragments containing the hinge region of proteolyzed IgG) have been found in synovial fluid from rheumatoid arthritis patients[53], breast carcinoma extracts[66], and associated with bacterial infections such as *Streptococcus pyogenes*[54] and *Pseudomonas aeruginosa*[76]. The presence of proteolyzed IgG associated with both cancerous cells and invasive microorganisms suggests that IgG proteolysis may be the basis of an immune evasion mechanism. Tumors and many microorganisms secrete proteases that cleave IgG molecules at defined sites in the hinge region[53,66], potentially resulting in inactivation or complete removal of the Fc region that normally mediates important host defense functions, such as ADCC, antibody-dependent cellular phagocytosis (ADCP), and CDC. Support for this *in vivo* defense mechanism was provided by *in vitro* experiments in which proteolysis of purified IgGs using physiologically relevant proteases targeting the hinge region (e.g., various human matrix metalloproteinases, GluV8 from *Staphylococcus aureus*, IdeS from *Streptococcus pyogenes*) led to complete loss of ADCC and CDC activities of a monoclonal antibody *in vitro* with no effect on antigen binding[66]. The loss of Fc-mediated functions was observed not only when the Fc was completely removed (both heavy chains cleaved in the hinge region), but even when only a single cleavage event took place (i.e., only one of the heavy chains was cleaved in the hinge region), and the Fc remained associated with the antigen binding domains. Importantly, addition of affinity-purified HAH autoantibodies reactive with $F(ab')_2$ fragments, but not intact IgG, was able to completely restore the lost ADCC and CDC activity of the proteolytically generated $F(ab')_2$ fragments (Fig. 2C), presumably by providing a surrogate Fc domain[6]. Thus, a possible *in vivo* function of HAH autoantibodies is to counter immune evasion strategies of tumors and invasive microorganisms by restoring key immune system defense mechanisms.

## IMPLICATIONS OF HAH AUTOANTIBODIES FOR USE OF THERAPEUTIC ANTIBODY FRAGMENTS

The prevalence of HAH autoantibodies in the normal human population has the potential to affect the efficacy of therapeutic monoclonal antibody fragments. The first recombinant monoclonal antibody product approved in the U.S. was abciximab, a chimeric Fab fragment generated by papain treatment of IgG. Its molecular target is the GPIIb/IIIa ($\alpha_{IIb}\beta_3$) integrin on the surface of human platelets. It was found that most humans have detectable reactivity to the papain cleavage site in the upper hinge region of human Fab fragments (i.e., HAH), including abciximab[75]. If pre-existing HAH autoantibodies bound to abciximab while it was bound to the platelet surface via GPIIb/IIIa during therapy, there was the potential for platelet clearance mediated by the Fc regions of HAH autoantibodies. In clinical studies, it was found that although abciximab-coated platelets could bind HAH autoantibodies *ex vivo*[77], there were few adverse clinical effects, including acute thrombocytopenia, that could be attributed to HAH autoantibodies in clinical trials[75]. Interestingly, the pretreatment HAH autoantibody reactivity tended to increase modestly after abciximab treatment, peaking at the earliest time points tested (1–3 weeks) compared to typical induced immune responses against the murine variable region of abciximab that peaked at 4–6 weeks following treatment[75], suggesting that this increased HAH autoantibody reactivity was unlikely to be a primary induced immune response and was perhaps indicative of an anamnestic response.

The low rate of acute thrombocytopenia seen in abciximab clinical trials (<2%)[78] may reflect the relatively low reactivity of HAH autoantibodies targeting the papain cleavage site in the upper hinge compared to cleavage sites in the lower hinge that generate $F(ab')_2$ fragments[6,57], as discussed previously. Support for this idea was provided by Yano and coworkers[79], who used a different anti-GPIIb/IIIa antibody, hC4G1, and its fragments, in a series of studies in cynomolgus monkeys. Following administration of the pepsin-generated $F(ab')_2$ fragment of hC4G1, severe thrombocytopenia was observed in five of 18 monkeys. Autoantibodies directed against the hinge region of the $F(ab')_2$ fragment were detected in most monkeys, and there was an inverse correlation between the level of the antihinge autoantibodies and platelet counts following $F(ab')_2$ administration, with the five monkeys exhibiting the highest level of autoantibodies having the most severe platelet depletion. In a separate study, administration of the Fab fragment of hC4G1 did not lead to thrombocytopenia in monkeys[80].

To avoid the potential for reactivity of future therapeutic antibody fragments with endogenous HAH autoantibodies, one strategy is to produce recombinant fragments choosing the C-terminal amino acid of the heavy chain to be a position that is nonreactive with endogenous HAH autoantibodies. Such nonreactive positions in the hinge were identified using a series of peptide analogs with free C-termini corresponding to each amino acid position across the IgG1 hinge[6].

## CONCLUSIONS

Although the existence of autoantibodies that bind to cryptic epitopes exposed by proteolysis of the IgG hinge region has been appreciated for some time, the origin and biological relevance of HAH autoantibodies remains unclear. However, several studies have defined the binding epitopes of HAH autoantibodies. These specificity studies suggested that there are multiple binding sites in both the upper and lower hinge region for HAH autoantibodies. Additionally, HAH autoantibodies have been identified with different isotypes. Taken together, these results suggest that there could be many different antihinge clones, including NAbs (with germline variable regions), as well as HAH autoantibodies that arose as a result of immune responses. Several biological mechanisms have been attributed to HAH autoantibodies, ranging from immunosuppressive effects to augmenting the host immune response. Molecular cloning of antihinge-specific autoantibodies from B cells should provide a useful tool to define the origin of HAH autoantibodies by variable region sequence analysis and help to elucidate the biological role of HAH autoantibodies with defined targets in hinge region.
